# NBP Relieves Cardiac Injury and Reduce Oxidative Stress and Cell Apoptosis in Heart Failure Mice by Activating Nrf2/HO-1/Ca^2+^-SERCA2a Axis

**DOI:** 10.1155/2022/7464893

**Published:** 2022-11-21

**Authors:** Zhongyu Wang, Yan Zhang, Lei Wang, Chun Yang, Hongliang Yang

**Affiliations:** ^1^Department of Cardiology, China-Japan Union Hospital of Jilin University, Changchun 130031, China; ^2^School of Public Health, Beihua University, Jilin 132033, China; ^3^Jilin Ginseng Academy, Changchun University of Chinese Medicine, Changchun 130117, China; ^4^College of Basic Medicine, Beihua University, Jilin 132033, China

## Abstract

Although heart failure (HF) has become one of the most fatal diseases in the whole world, there are fewer drugs for its treatment. Therefore, we focused on the protective effect of Dl-3-n-butylphthalide (NBP) on myocardial injury and oxidative stress in heart failure mice and further investigated the relationship with the Nrf2/HO-1/Ca^2+^-SERCA2a axis. *Methods*. C57BL/6J mice were divided into the sham group (Sham), heart Failure model group (HF), HF + NBP group (HN), HN + Nrf2 inhibitor (HNM), HN + Calmodulin-dependent protein kinase II (CaMKII) antagonist, KN93 (HNK). The HF mice model was prepared using abdominal aorta ligation. Mice's heart function was accessed by echocardiography. Hematoxylin-eosin staining and MASSON staining were used to identify myocardial injury; the cell apoptosis was determined by the TUNEL staining assay. The expression of oxidative stress-related proteins was detected by the ELISA assay. The reactive oxygen species and Nrf2 expression in heart tissue were observed with the immunofluorescence assay. SERCA2a, calmodulin, endoplasmic reticulum stress regulatory proteins, and Nrf2/HO-1 in mice' heart tissues were measured using Western blotting. *Results*. Moreover, NBP could significantly promote heart failure mice's heart function, relieve the injury and inhibit cell apoptosis. Meanwhile, it could reduce ERS injury of heart failure mice through increasing SERCA2a level and reducing Ca^2+^ influx. NBP was demonstrated to minimize CaMKII phosphorylation level and decrease cAMP-response element-binding protein phosphorylation level, suggesting NBP could also activate the Nrf2/HO-1 signaling pathway. *Conclusions*. We demonstrated that NPBs treatment promotes the cardiomyocyte's ERS and alleviates myocardial injury in heart failure mice, related to stimulating the Nrf2/HO-1 signaling pathway, regulating Ca^2+^-SERCA2a, and reducing Ca^2+^ influx.

## 1. Introduction

Heart failure (HF) is one of the most common diseases for hospital admissions worldwide [1]. HF is also a chronic, developing disease in which the heart cannot effectively pump enough blood to meet the metabolic need for blood and oxygen [2]. HF was usually accompanied by different complex clinically pathophysiological syndromes in circulation and pulmonary circulation congestion, seriously affecting the quality of life [3]. However, it is crucial to find effective prevention and treatment measures for ameliorating HF symptoms, reducing mortality, and promoting patient health.

The endoplasmic reticulum (ER) is the machine used for the processes of proteins including folding and transportation during many cellular events [4]. The endoplasmic reticulum homeostasis was affected during ischemia [5], hypoxia, drugs, or toxins [6], leading to the accumulation of misfolded proteins. ER is the major intracellular calcium store and is involved in several fundamental calcium signaling pathways [7]. The protein accumulation in the cavity or the imbalance of calcium ion (Ca^2+^) homeostasis leads to endoplasmic reticulum stress (ERS) [8]. As the endoplasmic reticulum stress response is severe or lasts for a long time, it may induce apoptosis and cause cell death [9]. Sarcoplasmic reticulum calcium ion transport ATPase-2a (SERCA2a) regulates the contraction and relaxation of cardiomyocytes by participating in the calcium ion transportation for maintaining calcium ion homeostasis and participating in regulation of endoplasmic reticulum stress response [10]. During endoplasmic reticulum stress, the reduction of SERCA2a activity is significantly reduced to cause an imbalance of calcium ion homeostasis in cardiomyocytes, which leads to cardiomyocyte contractile dysfunction and heart failure [11].

The nuclear factor erythroid 2-related factor 2/heme oxygenase-1 (Nrf2/HO-1) signaling pathway performed a crucial role in promoting heart functions and regulating myocardial remodel [12, 13].

During ERS, the degradation of Nrf2 in ubiquitination was remarkably reduced, and the function of Nrf2 was promoted [14]. The upregulation of Nrf2 could increase the HO-1 level and promote the antioxidant effect of cardiomyocytes via combining with angiotensin II receptor blocker (ARB), suggesting that Nrf2 is a factor of cardiomyocyte apoptosis [15, 16]. Therefore, it has a high value in clinical research for investigating the effective drug to inhibit cardiomyocytes' ERS response and keep the balance of myocardial calcium to enhance patients' heart functions and decrease heart failure-related mortality.

Dl-3-n-Butylphthalide (NBP) has been isolated from the seeds of *Apium graveolens* [17]. The FDA of China approved the NBP's application in ischemic stroke therapy in 2002 [18]. Meanwhile, many studies reported that NBP relieved the symptoms of ischemic stroke and aided in long-term recovery [19, 20]. However, the underlying mechanism of NBP improving heart failure is still not completely clear. Therefore, we focused on the therapeutic effect of NBP treatment on heart function and the relationship with ERS in heart failure mice and further discovered the underlying relationship with the Nrf2/HO-1 pathway.

## 2. Materials and Methods

### 2.1. Laboratory Animals and Grouping

Sixty C57BL/6J mice, all male and seven weeks old, were purchased from Beijing Huafukang Laboratory Animal Technology Co., Ltd. Mice were bred at 20–26°C, 40–70% humidity in the laboratory animal center of Jilin University. Mice were randomly divided into five groups (*n* = 15): sham operation group (SH), HF model group (HF), HF + NBP (HN), HF + NBP + CaMKII (KN93) (HNKC), HF + NBP + Nrf2 inhibitor (HNM). The animal experiment was approved by the Laboratory Animal Welfare and Ethics Committee of Jilin University (IACUC No.2019156).

### 2.2. Mice HF Model Preparation

The preparation of the mice HF model followed this protocol: Except for mice in the SH group, mice in other groups were performed with the constriction of the abdominal aorta assay to prepare HF mice models [21]. Firstly, mice were anesthetized using 1% pentobarbital sodium (45 mg/Kg) with intraperitoneal injection; then mice were put on a surgery platform with a heating pad to maintain the body temperature. The abdominal cavity was cut at the center of the abdomen for 2∼3 cm, the muscles were bluntly stripped, the abdominal aorta was exposed, a 7-gauge surgical needle was placed parallel to the abdominal aorta, and the aorta was ligated with 0-gauge silk thread. After that, the surgical needle was removed to confirm the aorta was unobstructed and the abdomen was closed. In the sham operation group, mice were anesthetized and the abdominal aorta of mice was isolated but not ligated. Mice were injected intraperitoneally with 50,000 U of penicillin daily for three days after the operation for infection prevention.

The mice in the SH group were administrated with the same amount of penicillin daily after operation for three days. After the surgery, mice in the HN group were treated with NBP (60 mg/kg) daily by intragastric administration. Besides, mice in the HNK were cotreated with the same amount (60 mg/kg) of NBP and CaMKII antagonist (KN93) with injection via tail vein postsurgery for 15 days. Meanwhile, mice of the HNM group were treated with an Nrf2 inhibitor (ML385, MCE, USA) before intragastric administration of NBP for 15 days.

### 2.3. Mice Heart Function Detection

Mice's heart function was detected by Cardiac ultrasound. M-mode echocardiography detection was performed for the examination. The measurement of the thickness of the left ventricular anterior wall (LVAW), left ventricular posterior wall (LVPW), and left ventricular end diastolic diameter (LVEDD) and left ventricular end-systolic (LVEDs), left ventricular ejection fraction (LVEF), and left ventricular fractional shortening (LVFS) were performed. These data were recorded for three cardiac cycles, and the average value was calculated as the final results.

### 2.4. Hematoxylin-Eosin (H&E) Staining

The formalin-fixed cardiac tissues were fixed in formalin solution. Mice tissues were gradually dehydrated in gradient ethanol, transparent in xylene, and embedded in paraffin. Mice heart tissue samples were cut into 4 *μ*m sections. Following that, the slices were stained with hematoxylin and eosin (H&E). Finally, the slices were observed under the microscope. All results were confirmed by at least 2 pathologists who were blind to animal grouping and treatment.

### 2.5. Masson Staining

The slices of mice heart tissue were deparaffinized and rehydrated with ethanol. Then the slices were stained with Regaud hematoxylin solution. Following that, slices were stained in Masson ponceau-acid solution for 15 min, then differentiated in 1% molybdenum phosphate acid. The sections were directly transferred to a decolorized blue aniline solution. Afterwards, sections were differentiated in 0.2% glacial acetic acid. The slices were dehydrated in 100% ethanol, rinsed in xylene and mounted in neutral resins. The slices were observed using a light microscope (Olympus, Tokyo, Japan).

### 2.6. TUNEL Staining

TUNEL assay kit (Roche Diagnostics, Mannheim, Germany) was used for cell apoptosis detection. The experiment procedure is followed with detailed instructions in the kit. The samples were observed and detected with a fluorescence microscope.

### 2.7. Immunofluorescence Assay (IFA)

Postdeparaffinized slices were submerged in hydrogen peroxide solution, rinsed in PBS, and put into a 0.1 M sodium citrate solution. After incubating with goat serum solution for 30 min, the slices were added with the probe of ROS and Nrf2 antibody and incubated overnight at 4°C. After washing in PBS, a fluorescent dye-labeled secondary antibody was added to the sections and incubated at 37°C for 30 min in darkness. DAPI dye was performed to stain the cell nucleus. After slices were mounted in mounting solution, the targeting proteins were observed using the fluorescence microscope.

### 2.8. ELISA

An ELISA Kit (ab108816, Abcam) was performed to detect the level of BNP, and an ELISA Kit (CEA485Ra, USCN, China) was also performed to examine the concentration of NT-proBNP in mice serum. In addition, the ELISA kits of superoxide dismutases (SOD) (ab65354, Abcam), malondialdehyde (MDA) (ab238537, Abcam), and catalase (CAT) (ab83464, Abcam) were also used according to the manufacturer's protocols.

### 2.9. Western Blotting

Heart tissue of mice was homogenized with RIPA buffer (CST, USA) at 15,000 rpm for 30 seconds and centrifugated for 15 min at 15,000 g at 4°C. The protein concentration in the supernatant was determined by BCA assay. 30 *μ*g total proteins was loaded and separated in 10–12% SDS-PAGE gels under electrophoresis and transferred to PVDF membranes. After blocking in 5% skim milk, PVDF membranes were separately incubated overnight at 4°C with primary antibodies against Bcl-2 (3498, CST, USA), Bax (5023, CST, USA), CaMKII (13–7300, Abcam, USA), p-CaMKII (12716, CST, USA), cAMP-response element-binding (CREB) (4820, CST, USA), p-CREB (9198, CST, USA), SERCA2a (4388, CST, USA), dihydropyridine receptor (ab232983, Abcam, USA), Cav1.2 (ab84814, Abcam, USA), Caspase-12 (2202, CST, USA), Nrf2 (12721, CST, USA), HO-1 (43966, Abcam, USA), thioredoxin (Trx-1) (2298, CST, USA), GRP78 (ab21685, Abcam, USA), and GAPDH (51742, CST, USA) as the internal control. Then, PVDF membrane was incubated with diluted HRP-conjugated goat anti-rabbit secondary antibody (1 : 1000,5597, CST, USA) for one hour. The protein bands were visualized with SuperSignal™ West Pico PLUS Chemiluminescent Substrate (CAT#3480, Thermo Fisher Scientific, USA). The quantification of protein bands was determined by ImageJ (National Institutes of Health, Bethesda, MD). The grey results were showed as relative density after correction with the expression of GAPDH.

### 2.10. Statistical Methods

These results were analyzed by Graphpad Prism 8.0 and represented as mean ± SD. To determine the statistically significant difference, an unpaired Student's *t*-test was used for two-group comparisons; one-way analysis of variance (ANOVA) was performed, followed by multiply comparisons. A *P*value less than 0.05 was considered significant.

## 3. Results

### 3.1. The Protective Effects of NBP on HF Mice Heart

**Figure 1:**
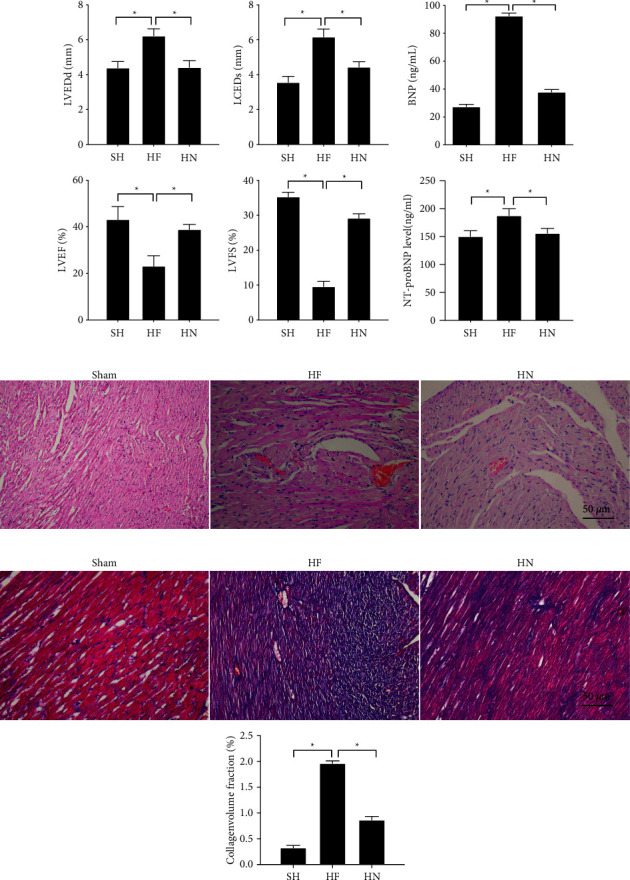
NPB promoted heart function of heart failure mice and relieved the myocardial injury. (a) The echocardiographic results; (b) The ELISA results of BNP level and NT-proBNP level; (c) pathological damages examined by HE staining (scale bar = 50 *µ*m); (d) Masson staining (scale bar = 50 *µ*m); ‘^*∗*^'*P* < 0.05. *n* = 15.

We investigated the NBP effect on HF mice; we successfully prepared the mice HF model by constricting the abdomen aorta. The results showed that in the HF group, the left ventricular end-systolic (LVDs) and diastolic (LVDd) were significantly increased, LVEF and LVFS were reduced (Figure 1(a), *P* < 0.05), and the level of heart failure biomarkers BNP and NT-proBNP in plasma were both increased (Figure 1(b), *P* < 0.05). Compared with mice in the sham group, the arrangement of myocardial fiber cells is disordered, myocardial fibers are rupture in the HF group (Figure 1(c), *P* < 0.05). These results confirmed that the HF mice model was established successfully. Through NBP administration, the phenotypes in the HF mice model were all reversed (Figure 1(a)–1(c), *P* < 0.05). Our data showed that NBP could significantly relieve the myocardial injury in HF mice and enhance the left ventricular systolic and diastolic function.

### 3.2. Treatment of NBP Promotes Pathological Results

**Figure 2:**
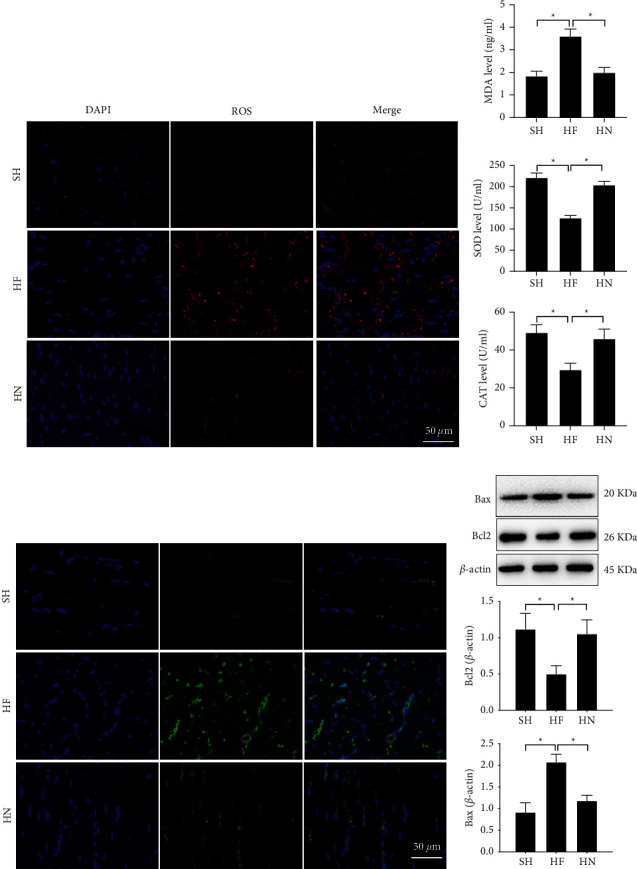
NPB alleviated oxidative stress injury and decreased the apoptosis of cardiomyocytes of heart failure mice. (a) The levels of ROS in mice was examined by IFA (scale bar = 20 *µ*m); (b) The level of MDA, SOD, and CAT of plasma examined by ELISA assay; (c) The TUNEL staining results demonstrated the rat myocardial apoptosis (scale bar = 20 *µ*m); (d) Bax and Bcl-2 identified using Western blotting; ‘^*∗*^'*P* < 0.05. *N* = 15.

To investigate the relationship between NBP and oxidative stress injury in the myocardium, firstly, we observed the accumulation of ROS in HF mice (Figure 2(a)), interestingly NBP administration significantly reduced the content of ROS, decreased the MDA level in plasma and increased the expression of SOD and CAT (Figure 2(b)). Moreover, myocardial apoptosis rate was slow significantly promoted in the HF group (Figure 2(c)); Bax was downregulated during heart failure mice. Furthermore, the antiapoptotic factor Bcl-2 was decreased (Figure 2(d)). These pathological damages were relieved after NBP treatment on HF mice, suggesting that NBP reduces oxidative stress injury and decreases cardiomyocyte apoptosis.

### 3.3. The Effect of NBP Is Associated with ERS Homeostasis

**Figure 3:**
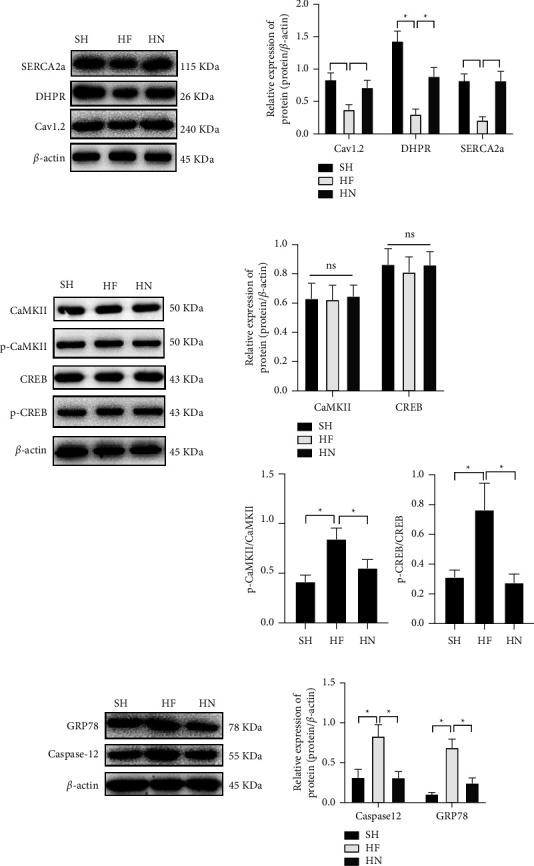
NPB treatment could enhance Ca^2+^-SERCA2a and inhibit ERS response in HF mice. (a) Western Blotting results showed the expression of SERCA2a, calcium-regulated proteins; (b) Western Blotting results showed the level phosphorylation of CaMKII and CREB; (c) Western Blotting results showed the ERS-associated regulatory proteins. ‘^*∗*^'*P* < 0.05. *n* = 3.

The over-activation of ERS was reported to stimulate sarcoplasmic reticulum dysfunction of Ca^2+^ uptake, causing intracellular Ca^2+^ burden, inducing cardiomyocyte apoptosis, leading to myocardial contractile and diastolic function. Maintaining intracellular Ca^2+^ homeostasis is related to SERCA2a expression. To explore the regulatory mechanism of SERCA2a under NBP treatment, we demonstrated that the expression of SERCA2a was reduced in HF mice (Figure 3(a)), leading to Ca^2+^ transport disorder, resulting in inhibited levels of calmodulin, DHPR, and Cav1.2 (Figure 3(a)). The overloading of Ca^2+^ stimulated the binding with CaMKII to enhance the phosphorylation of CaMKII and CREB (Figure 3(b)), meanwhile, the levels of GRP78 and Caspase-12, ERS related proteins, were upregulated in HF mice (Figure 3(c)), suggesting that ERS can cause the degradation of SERCA2a, resulting in decreased cardiac diastolic function. After NBP treatment, SERCA2a was upregulated, the calcium-regulated proteins were activated, resulting in the inhibition of the phosphorylation of CaMKII and CREB. Meanwhile, the ERS injury was alleviated as GRP78 and Caspase-12 were downregulated, suggesting that NBP inhibits ERS via stimulating Ca^2+^-SERCA2a signaling pathway in HF mice.

### 3.4. NBP Treatment Could Motivate the Nrf2/HO-1 Pathway

**Figure 4:**
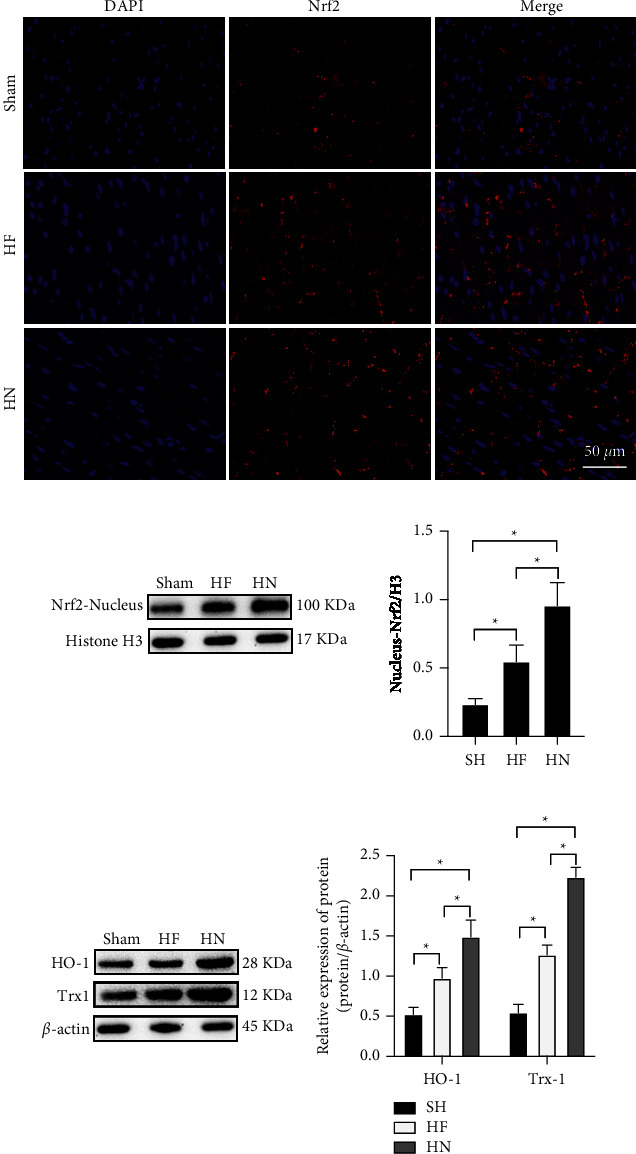
NPB treatment stimulates Nrf2/HO-1 pathway in heart failure mice. (a) The results of IFA showed the Nrf2 expression in mice's myocardial nucleus and cytoplasm detected by IFA (scale bar = 20 *µ*m); (b) The result of western blot demonstrated the expression of Nrf2, HO-1, and Trx1) ‘^*∗*^'*P* < 0.05. *n* = 3.

Maintaining equilibrium between oxidative and antioxidative components in the ER is important in regulating the oxidative stress response in cardiomyocytes. As in maintaining the ER balance, Nrf2, one of the most important regulators in ER balance, was demonstrated to transfer into the cell nucleus in the HF model (Figure 4(a)). As the result of that, the release of HO-1 and Trx1 were promoted during heart failure (Figure 4(b)). After the administration of NBP, Nrf2 (Figure 4(a) and 4(b)), the high cytoplasmic expression of Nrf2 promoted the further increased expressions of its downstream proteins, speculating that NBP improves the ERS through activating the Nrf2/HO-1 pathway of Heart Failure mice.

### 3.5. CaMKII Antagonist Inhibits the Benefits of NBP on Myocardium in HF Mice

**Figure 5:**
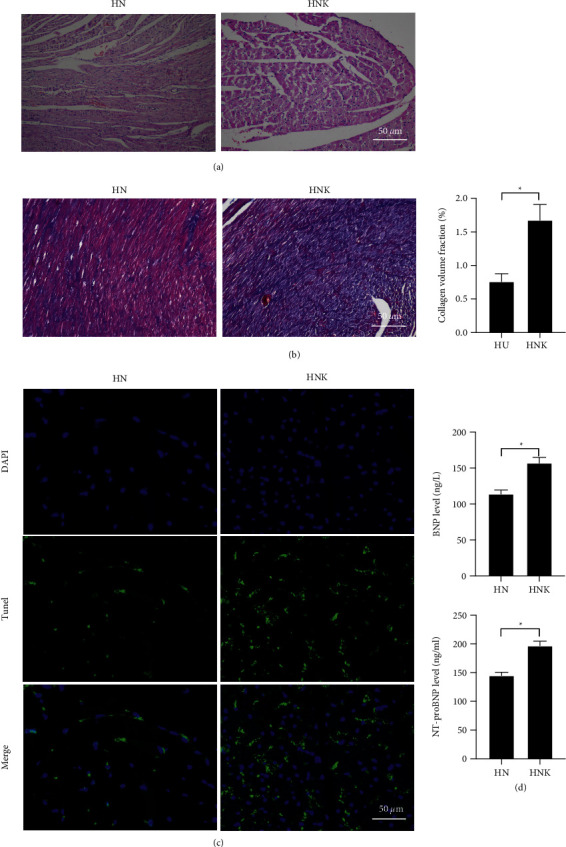
The therapeutic effect of NPB treatment on myocardium in HF mice was partially reversed by CaMKII antagonist. (a) The results of HE staining showed the pathological changes in myocardial tissues (scale bar = 50 *µ*m); (b) The results of Masson staining showed the tissue fibrosis (scale bar = 50 *µ*m); (c) The results of TUNEL staining show the myocardial apoptosis rate (scale bar = 20 *µ*m); (d) The natriuretic peptide levels were examined by ELISA; ‘^*∗*^'*P* < 0.05. *n* = 3.

We also investigated the detailed mechanism of NBP treatment on calcium channels, thus the CaMKII antagonist, KN93, was administered in HF mice before NBP treatment. Interestingly, the protective effects of NBP on myocardium in HF mice were partially suppressed. The results demonstrated that KN93 treatment could partially block the therapeutic effect of NBP on myocardial injury (Figure 5(a)) and increase the percentage of collagen volume fraction (Figure 5(b)) compared with mice in HN group. Moreover, KN93 also increased the apoptosis rate (Figure 5(c)) and promote the expression of BNP and NT-proBNP (Figure 5(d)). These results demonstrated that the CaMKII antagonist, KN93 could partially block the effect of NBP.

### 3.6. Nrf2 Inhibitor Suppresses the Activation of Ca^2+^-SERCA2a

**Figure 6:**
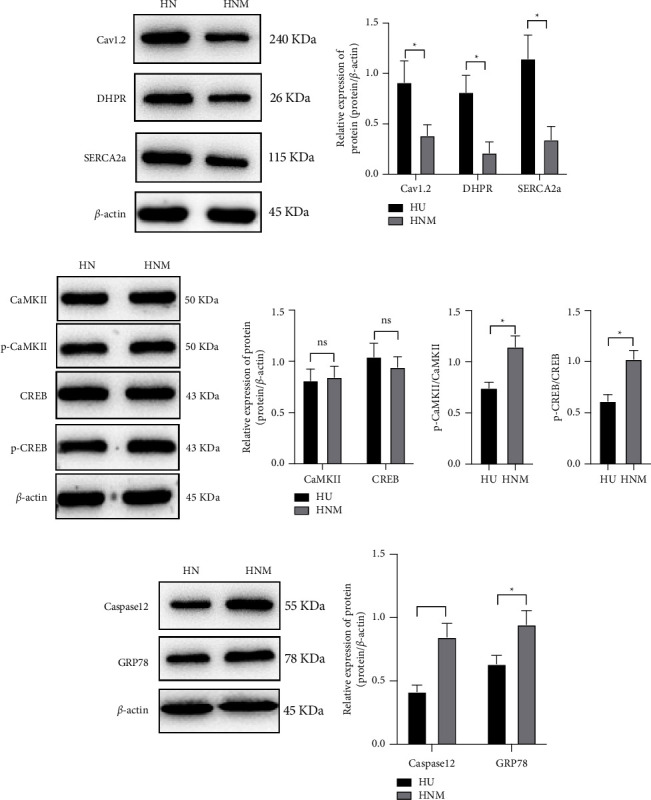
Nrf2 inhibitor blocks the activation of Ca^2+^-SERCA2a under NBP treatment. (a) Western blotting results showed the expression of SERCA2a, calcium-regulated proteins; (b) Western blotting demonstrated the phosphorylation level of CaMKII and CREB; (c) Western Blotting results showed the ERS-associated regulatory proteins.‘^*∗*^'*P* < 0.05. *n* = 3.

We also investigated the relationship of the Nrf2/HO-1 pathway with the Ca^2+^/CaMKII/CREB pathway; ML385, an Nrf2 inhibitor, was used to treat HF mice before NBP administration. The Ca^2+^ overload was blocked after ML385 treatment. Moreover, the expression of SERCA2a (Figure 6(a)) and calcium regulatory proteins (Figure 6(b)) were downregulated in the HNM group. Therefore, the phosphorylation of CaMKII and CREB was promoted. Nrf2 inhibitor could upregulate the ERS regulatory factors (Figure 6(c)). Our data demonstrated that the protective role of NBP on HF mice depends on the activation of the Nrf2/HO-1/Ca^2+^-SERCA2a axis.

## 4. Discussion

NBP is an important compound, which is extracted from celery seeds [18]. NBP can also be obtained using chemical synthesis methods. NBP has become an emerging therapeutic drug. A large number of cell and animal studies have shown that NBP can protect the brain from acute ischemic damage, reduce the area of cerebral infarction, inhibit platelet aggregation, and reduce neuronal apoptosis and oxidative damage [18]. Chinese researchers conducted two double-blind, randomized, multicenter trials, demonstrating that NBP exerted therapy effects on stroke. Due to its safety and effectiveness, NBP was approved by the US Food and Drug Administration in 2002 [18]. Recently, NBP was reported to play a preventive role in acute myocardial infarction. However, it is still unclear whether NBP has a protective effect on chronic heart failure. Therefore, in this study, the mice model of chronic heart failure was constructed by surgery and postsurgery exercise. After the HF mice model was successfully established, NBP was given to explore the effects of NBP on heart function, oxidative stress, cardiomyocytes, and apoptosis in chronic heart failure mice.

Chronic heart failure is characterized by heart muscle weakness and diastolic dysfunction, as well as a decreased cardiac output. The heart was unable to meet the needs of the body's cell metabolism and venous blood return was blocked [22]. The changes in hemodynamics and neurohumoral could lead to various symptoms and signs [23]. Epidemiological data reported that the survival rate over five years period of heart failure patients is close to that of cancer, and a total of 40% of cardiovascular deaths are caused by heart failure [24]. Oxidative stress imbalance and cardiomyocyte apoptosis are the main pathological manifestations of chronic heart failure. Mitochondrial dysfunction is an important process during heart failure, leading to a large amount of reactive oxygen species released, inducing the production of MDA and SOD, leading to cardiomyocyte energy metabolism disorder. And the energy metabolism dysfunction of the cardiomyocyte reduced the contractile function, eventually leading to cardiomyocyte apoptosis. In this study, NBP was shown to improve the effect of the HF mice model on the systolic and diastolic heart function, reduce the cardiac muscle injury, alleviate myocardial fibrosis, reduce cardiomyocyte apoptosis, and ameliorate the oxidative stress damage caused by endoplasmic reticulum stress. These results demonstrated that NBP regulates calcium channels to perform the cardioprotective functions.

Heart failure is caused by a dysfunctional calcium regulation of the cardiomyocyte in the ER [25]. Ca^2+^ is the second messenger during cardiomyocyte excitation-contraction coupling (ECC). The calcium cycle mainly includes two main processes: sarcoplasmic reticulum (SR) calcium release and calcium recapture [25]. After the ECC process is over, the removal of Ca^2+^ from cardiomyocytes' cytoplasm is caused by calcium pump SERCA2a to reuptake Ca^2+^ back to the SR. As HF occurs, the downregulation of SERCA2a indicates that Ca^2+^ influx causes intracellular calcium overload, stimulating Ca^2+^ binding to CaMKII, leading to phosphorylation of the substrate of CaMKII, promoting its autophosphorylation, and the activated CaMKII directly phosphorylates CREB and binds with a specific sequence of cyclic adenosine phosphate response elements, recruiting RNA polymerase II, to form a transcription complex to control gene transcription [10, 11]. In this study, drug treatment could significantly enhance SERCA2a expression in the HF mice model, thereby activating the calmodulin DHPR and Cav1.2 while reducing the CaMKII and CREB phosphorylation. Undertreatment with CaMKII antagonist, the regulative effect of NBP on Ca^2+^-SERCA2a is inhibited, and the myocardial protection of the HF mice model is inhibited, whereas suggesting that NBP treatment activates Ca^2+^-SERCA2a to promote myocardial calcium and protect the myocardial tissue in HF mice, which is associated with ERS.

When the Ca^2+^-SERCA2a homeostasis of cardiomyocytes is destroyed, the oxidation system and antioxidant system lose their homeostatic balance. The oxidation capacity exceeds its antioxidant capacity, causing abnormal high expression of some proteins such as GRP78 and caspase-12, which affects their completion of normal proteins [26]. Nrf2, as a core transcription factor that resists oxidative damage, can induce cell damage and cell dysfunction, such as necrosis or pyrolysis [27]. Studies have shown that the inhibition of Nrf2 can aggravate myocardial oxidative stress and apoptosis, leading to cardiac dysfunction. However, upregulating Nrf2 promoted the left ventricular function in mice with heart failure and reduced cardiac hypertrophy [28–30]. In our research, through stimulation of ERS, Nrf2 performed nuclear translocation and increased in the nucleus. After entering the cell nucleus, Nrf2 usually combines with another transcription CREB and induces the transcription process, which is consistent with the promotion of CaMKII and CREB phosphorylation in HF mice. As mice were treated with the Nrf2 inhibitor, the regulation function in Ca^2+^-SERCA2a homeostasis by NBP was inhibited. However, there are still some limitations in this study. We should examine the protective effect of NBP on other larger vertebrates with heart failure disease to fully illustrate its role. Moreover, the directly binding or regulating proteins of NBP administration need to be further investigated to discover the detailed molecular mechanisms. Based on these results, we concluded that NBP could protect the myocardium in heart failure and improve the ERS of cardiomyocytes by activating the Nrf2/HO-1/Ca^2+^-SERCA2a axis. This presented result demonstrated that NBP has a promising application in heart failure therapy, which may bring about favorable recovery of HF patients.

## Data Availability

The datasets used and analyzed during the current study are available from the corresponding author upon reasonable request.
